# A hydrophobic anchor mechanism defines a deacetylase family that suppresses host response against YopJ effectors

**DOI:** 10.1038/s41467-017-02347-w

**Published:** 2017-12-19

**Authors:** Marco Bürger, Björn C. Willige, Joanne Chory

**Affiliations:** 1 Plant Biology Laboratory, Salk Institute for Biological Studies, 10010 North Torrey Pines Road, La Jolla, CA 92037 USA; 2 Howard Hughes Medical Institute, Salk Institute for Biological Studies, 10010 North Torrey Pines Road, La Jolla, CA 92037 USA

## Abstract

Several *Pseudomonas* and *Xanthomonas* species are plant pathogens that infect the model organism *Arabidopsis thaliana* and important crops such as *Brassica*. Resistant plants contain the infection by rapid cell death of the infected area through the hypersensitive response (HR). A family of highly related α/β hydrolases is involved in diverse processes in all domains of life. Functional details of their catalytic machinery, however, remained unclear. We report the crystal structures of α/β hydrolases representing two different clades of the family, including the protein SOBER1, which suppresses AvrBsT-incited HR in *Arabidopsis*. Our results reveal a unique hydrophobic anchor mechanism that defines a previously unknown family of protein deacetylases. Furthermore, this study identifies a lid-loop as general feature for substrate turnover in acyl-protein thioesterases and the described family of deacetylases. Furthermore, we found that SOBER1’s biological function is not restricted to *Arabidopsis thaliana* and not limited to suppress HR induced by AvrBsT.

## Abstract (web summary)

A subset of α/β hydrolases is known to suppress the pathogen-triggered hypersensitive response (HR) in plants, but their mechanism of action remains unclear. The authors present two crystal structures and functional analyses of these enzymes, showing that HR is suppressed by a previously unknown family of deacetylases.

## Introduction

Pathogenic *Pseudomonas* and *Xanthomonas* strains pose a big threat to agriculture, with the two groups being responsible for a plethora of bacterial plant diseases. The basis of their pathogenicity is the injection of effector proteins into the host using a type III secretion system (T3SS) to remodel plant immune response^1,2^. Though few effector proteins are conserved among pathogens that infect hosts from different kingdoms, the YopJ effector family of gram-negative bacteria has been well characterized in a number of different pathogens. YopJ originally derives its name from *Yersinia pestis*, the causative agent of bubonic plague, and from *Yersinia pseudotuberculosis*, which causes Izumi fever in humans^3,4^. Though its putative structural fold suggests the protein being a cysteine protease, it has been biochemically established that YopJ is an acetyltransferase^5^. In mammals, acetylation by YopJ suppresses immune signaling through targeting kinases within the MAPK pathway. Acetyltransferase activity has also been reported for other members of the family, among them HopZ1a from *Pseudomonas syringae* ^6,7^ and AvrBsT from *Xanthomonas campestris* pv *vesicatoria* (Xcv)^8,9^. HopZ1a interferes with cytoskeleton stability and jasmonate signaling, as suggested by its reported acetylation of tubulin and JAZ proteins^7,10^. AvrBsT has been shown to acetylate ACIP1, a microtubule-associated protein that is involved in pathogen-associated molecular pattern (PAMP) triggered immunity (PTI), as well as AvrBsT-induced effector-triggered immunity (ETI)^9^. In plants, ETI is often associated with the hypersensitive response (HR), leading to rapid cell death of the infected region and preventing further spread of the pathogen^11,12^. The bacterial counter-strategy is to impede such response by suppression of HR, which happens through other effector proteins and intrinsic plant components^13^. The putative plant phospholipase AtSOBER1 was reported to suppress AvrBsT-elicited HR in *Arabidopsis thaliana* through its suggested phospholipase A_2_ (PLA_2_) activity. AtSOBER1 has thus been proposed as a negative regulator of phosphatidic acid accumulation, which occurs upon AvrBsT activity during infection, suggesting AvrBsT and AtSOBER1 are involved in lipid homeostasis^14^. However, the reported in vitro PLA_2_ activity of AtSOBER1 was compared to *Saccharomyces cerevisiae* acyl-protein thioesterase 1, an enzyme with a reportedly poor reaction rate (*K*_M_ = 2.1 mM) on lysophosphatidylcholine, a generic PLA_2_ substrate^15^. In addition, an earlier study demonstrated AtSOBER1’s preference for short acyl chain *para*-nitrophenyl esters^16^. The suggested link between AtSOBER1 and the group of acyl-protein thioesterases is rather vague. The latter are mainly known from their human isoforms APT1 and APT2, which catalyze the depalmitoylation of the oncogene Ras^17^. Both APTs and AtSOBER1 are members of the large group of α/β hydrolases and share a sequence identity of 33%. Nevertheless, α/β hydrolases, although sharing a common fold, can develop quite distinct biochemical activities due to the high flexibility of their core architecture. Their protein fold allows for insertions, deletions and a relatively flexible position of the catalytic triad residues, which are located on loops^18^. The biological significance of SOBER1 activity in plant species other than *Arabidopsis* and in the context of other YopJ effector proteins is unknown. Furthermore, AtSOBER1’s enzymatic nature is uncertain, because only limited information can be obtained by sequence homology and inconsistent results were published. Therefore, we conducted a thorough structural, biochemical, and in vivo study that involves several members of the esterase family. We solved the crystal structure of AtSOBER1 and carried out biochemical and physiological studies of several AtSOBER1 related proteins suggesting deacetylation as the molecular basis of HR suppression against YopJ effectors. In addition, we identified a loop segment acting as a lid structure in acyl-protein thioesterases, which turns out to be the major factor determining product turnover in this class of enzymes.

## Results

### Crystal structures of AtSOBER1 and ZmB6T1C9

**Fig. 1:**
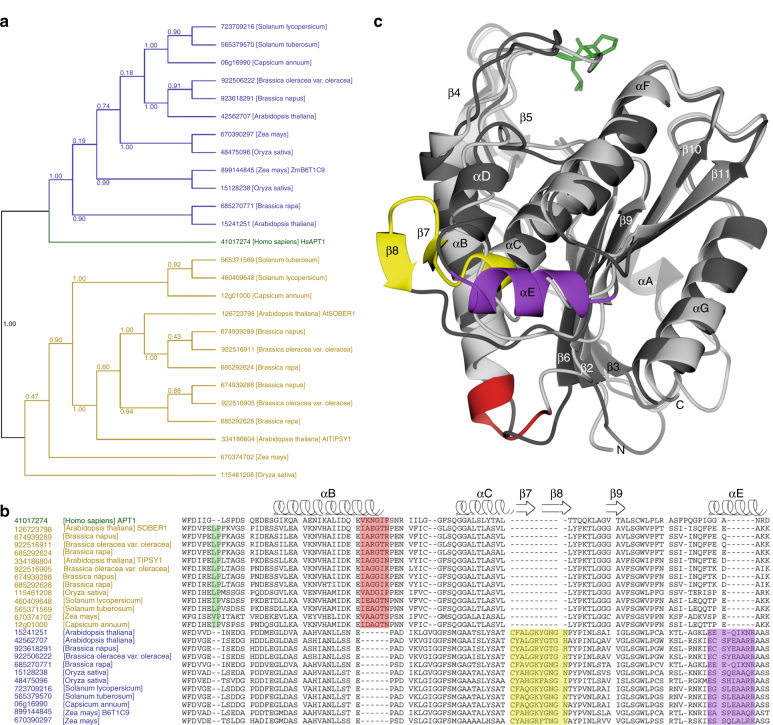
Phylogenetic and structural analysis of plant hydrolases reveals two major protein clades. **a** Phylogenetic tree of related plant hydrolases. **b** Multiple sequence alignment highlighting conserved sequence blocks within the clades. **c** Superimposition of AtSOBER1 (gray) and ZmB6T1C9 (black) showing the structural elements encoded by those blocks in the same colors

A phylogenetic analysis of plant acyl-protein thioesterases reveals two major clades featuring distinct and conserved blocks (Fig. 1a, b). To elucidate the molecular features encoded by these conserved elements, we solved the crystal structures of *Arabidopsis thaliana* SOBER1 and *Zea mays* B6T1C9, each representing a different clade in the phylogenetic tree (Fig. 1c). Both proteins fold into a canonical α/β hydrolase architecture and superpose with a root-mean-square deviation (RMSD) of atomic positions of 1.54 Å over 210 amino acid residues. They have human acyl-protein thioesterase 1 (pdb code 1fj2)^19^ and human acyl-protein thioesterase 2 (pdb code 5syn)^20^ as closest structural relative with an RMSD of 1.8 Å for AtSOBER1 and 1.6 Å for ZmB6T1C9 over 210 amino acid residues, respectively, as reported by a Dali search^21^. Most differences between the two proteins encode for minor structural features, such as an elongated helix and an additional beta strand. Furthermore, a two-amino acid insertion into a loop segment results in a functional alteration of the substrate binding site, splitting the protein family into two different classes of hydrolases.

### *Zea mays* B6T1C9 is a *bona fide* acyl-protein thioesterase

**Fig. 2:**
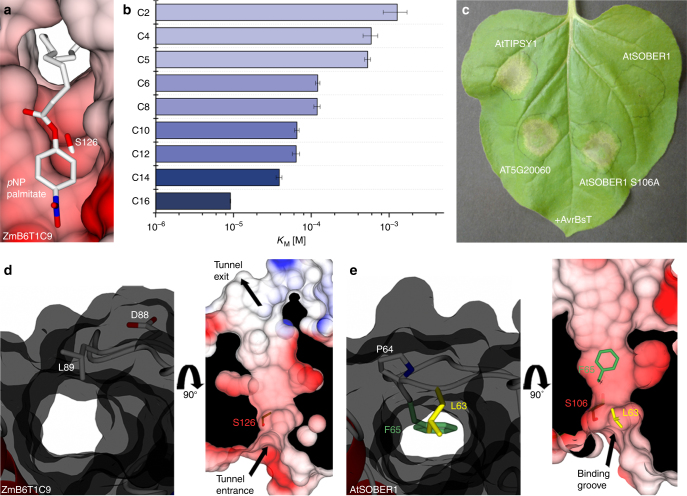
ZmB6T1C9 has an open substrate tunnel while the tunnel of SOBER1 is blocked. **a** Tunnel architecture of ZmB6T1C9 with docked *p*NP-palmitate, displaying the continuous tunnel through the protein and the position of catalytic serine S126. **b** *K*_M_ values of different *para*-nitrophenyl esters from *p*NP acetate (C2) through *p*NP-palmitate (C16) to ZmB6T1C9/ZmAPT2. Data were measured in triplicates and error bars indicate standard deviation. **c** Transient co-expression of AtSOBER1 (wild type and enzymatic dead mutant), AtTIPSY1 and AT5G20060 (closest *Arabidopsis* homolog of ZmB6T1C9) with avrBsT in *N. benthamiana*. **d** Open tunnel architecture in ZmB6T1C9. **e** Blocked tunnel architecture in AtSOBER1 and location of tunnel blocking residues L63 and F65

Classical acyl-protein thioesterases (APTs) feature an active site accessible to the solvent and adjacent to a hydrophobic tunnel for accommodation of fatty acids attached to their protein substrates. The crystal structure of ZmB6T1C9 reveals exactly such architecture with a continuous tunnel stretching through the entire protein, allowing for insertion of long fatty acids and their hydrolysis by the catalytic triad of the enzyme (Fig. 2a). We determined Michaelis–Menten parameters for hydrolysis of a series of *p*-nitrophenyl esters and found stronger affinities for longer fatty acid chains with *p*NP palmitate binding almost 200 times stronger to the protein compared to *p*NP acetate (Fig. 2b). We therefore conclude that the phylogenetic clade containing ZmB6T1C9 represents canonical acyl-protein thioesterases (APTs). Because of its RMSD being closer to human APT2, we assigned ZmB6T1C9 *Zea mays* as acyl-protein thioesterase 2 (ZmAPT2).

It was suggested that the potential lipase activity of AtSOBER1 is responsible for suppression of AvrBsT-induced HR^14^. Therefore, we were interested to test if members of the clade containing ZmB6T1C9/ZmAPT2 can execute a similar biological function. For this purpose, we used *Agrobacterium*-mediated transient expression in *Nicotiana benthamiana* to analyze AvrBsT activity and its suppression independent of other effector proteins. As previously reported, AtSOBER1 repressed AvrBsT-elicited HR, while its paralog (which we named AtTIPSY1) did not^16^. Furthermore, the closest *Arabidopsis* homologs of ZmB6T1C9/ZmAPT2 (AT5G20060) could not compromise the induced HR as well (Fig. 2c, Supplementary Fig. 1a), which indicates that a preference for long acyl chains is not sufficient to suppress AvrBsT-triggered HR.

### A conserved insertion in SOBER1 blocks the substrate tunnel

Similar to the crystal structure of ZmB6T1C9/ZmAPT2, we discovered a substrate tunnel in the AtSOBER1 protein, though severely impaired by a two amino acid insertion (LP63-64) into the loop that constitutes the tunnel lid (Fig. 2d, e, Supplementary Fig. 2). We found one insertion (L63) to directly block the entrance to the tunnel. The insertion of the second-amino acid (P64) pushes the following residue (F65) into the tunnel, which is positioned in the middle of the hydrophobic tunnel and could act as a hydrophobic anchor for the lid. The two amino acid insertion is conserved within the entire family clade (Fig. 1b), well aligning with the leucine at the tunnel entrance and the phenylalanine in the center of the tunnel, the latter of which is in most cases a phenylalanine or a leucine. To investigate the enzymatic consequences of the insertion and the mechanistic architecture of the lid, we subsequently conducted a series of Michaelis–Menten experiments.

### Leu63 provides a surface for deacetylation

**Fig. 3:**
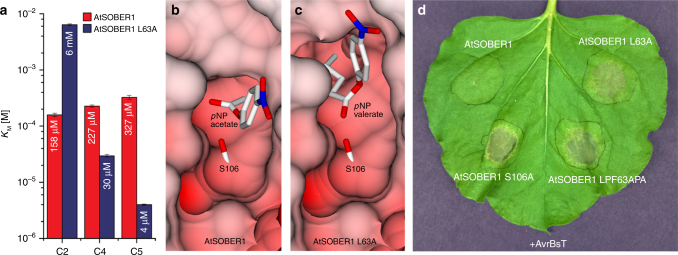
Altering SOBER1 protein surface changes substrate specificity and biological function. **a** *K*_M_ values of AtSOBER1 wt and L63A to *p*NP acetate (C2), *p*NP butyrate (C4), and *p*NP-valerate (C5). Data were measured in triplicates and error bars indicate standard deviation. **b** Docked *p*NP acetate into the catalytic site of wild type AtSOBER1. **c** Docked *p*NP-valerate into the catalytic site of AtSOBER1 L63A. **d** Transient co-expression of AtSOBER1 (wild type and mutant variants) with avrBsT in *N. benthamiana*

We obtained Michaelis–Menten parameters for hydrolysis of different *p*-nitrophenyl esters by AtSOBER1. In contrast to *Zea mays* acyl-protein thioesterase 2 and in agreement with Cunnac et al. (2007), *Arabidopsis* SOBER1 displayed a clear preference for *p*NP acetate (Fig. 3a). In contrast to Kirik and Mudgett (2009), we were unable to detect any phospholipase 2 activity of AtSOBER1 in vitro (Supplementary Fig. 3). To test for the importance of L63 for deacetylation, we constructed a L63A mutant of AtSOBER1 and analyzed its structural and biochemical properties. While wild type AtSOBER1 features a shallow binding groove for the acetate, with a protein surface provided by the side chain of L63 (Fig. 3b), the crystal structure of the L63A mutant showed a massively changed surface, with the hydrophobic tunnel now opened up right behind the active site (Fig. 3c). While the mutant protein was still an active enzyme, we discovered that its substrate specificity was now reversed: We examined the mutant in kinetic studies and found its *K*_M_ to *p*NP acetate weakened by about 40-fold compared to wild type. In addition, longer substrates were able to enter the tunnel, as demonstrated by higher affinities to *p*NP butyrate (C4), valerate (C5) and hexanoate (C6) (Fig. 3a). Furthermore, we tested the L63A mutant in tobacco leaves and found the protein to be compromised in AvrBsT-elicited HR repression. A more severe mutation in the hydrophobic tunnel (LPF63APA) led to a complete loss of HR suppression (Fig. 3d, Supplementary Fig. 1b).

### Ability to suppress HR is not determined by protein termini

**Fig. 4:**
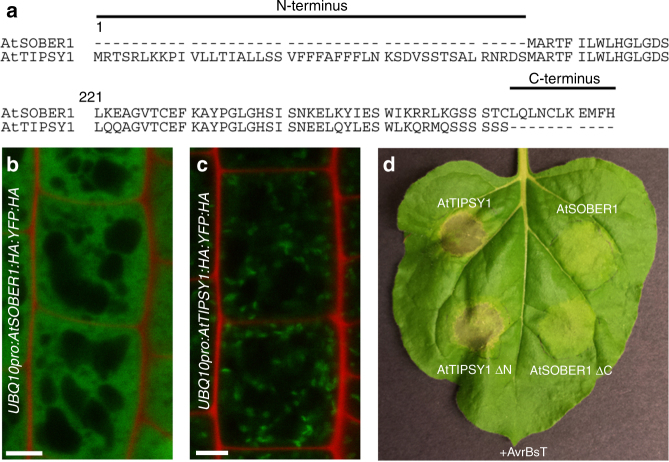
Suppression of HR is not determined by the protein termini of SOBER1 and TIPSY1. **a** Terminal sequences of both *Arabidopsis* proteins. **b**, **c** Localization of YFP-tagged AtSOBER1 (**b**) and AtTIPSY1 (**c**) in epidermal root cells of transgenic *Arabidopsis* seedlings. YFP signals are depicted in green, while propidium iodide, which was used for counterstaining, is shown in red. **d** Transient co-expression of AtSOBER, AtTIPSY1 and their truncated mutant variants with avrBsT in *N. benthamiana*. Scale bar = 5 µm

In *Arabidopsis thaliana*, SOBER1 and TIPSY1 are arranged in tandem and are therefore likely the result of a gene duplication event. The most obvious differences between both protein sequences are their termini. While SOBER1’s C-terminus is slightly extended, TIPSY1’s N-terminus comprises 45 additional amino acids (Fig. 4a). We analyzed transgenic *Arabidopsis* seedlings expressing fluorescently tagged versions of both paralogs via confocal microscopy and used root cells to ease localization. As reported for AvrBsT in *N. benthamiana* leaves^22^, AtSOBER1 was localized in the cytoplasm and the nucleus (Fig. 4b, Supplementary Fig. 4a). AtTIPSY1 could be found in both compartments as well, but was mainly localized in vesicle-like structures (Fig. 4c, Supplementary Fig. 4b). Since AtTIPSY1’s N-terminus could be a potential signaling sequence responsible for its compartimentation, we tested an N-terminally truncated mutant variant in tobacco. Like the wild type protein, it was unable to suppress AvrBsT-induced HR. Furthermore, loss of the C-terminus of AtSOBER1 did not alter its tested biological function, indicating that other protein characteristics are responsible for the differences between both paralogs (Fig. 4d, Supplementary Fig. 1 c).

### A lid-loop determines catalytic efficiency

**Fig. 5:**
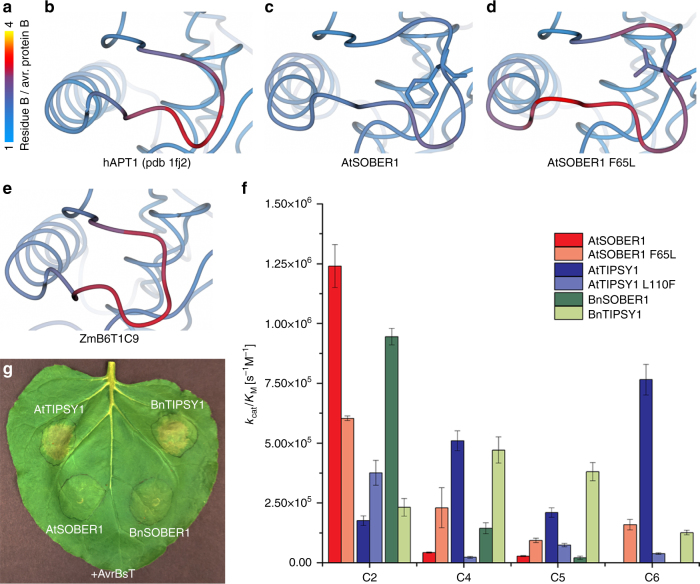
Enzymatic and biological properties are conserved between AtSOBER1 and BnSOBER1. **a**–**e** Lid-loop temperature factors of wild type AtSOBER1, AtSOBER1 F65L and of the acyl-protein thioesterases ZmB6T1C9 and human APT1. **f** Catalytic efficiencies of wild type and mutant SOBER1/TIPSY1 from *Arabidopsis thaliana* and *Brassica napus* on different *para*-nitrophenyl esters. Data were measured in triplicates and error bars indicate standard deviation. **g** Transient co-expression of SOBER1/TIPSY1 from *Arabidopsis thaliana* and *B. napus* with avrBsT in *N. benthamiana*

To investigate the structural importance of the loop, which constitutes the tunnel lid, we examined temperature factors of all structures solved in this study and of the published structure of human acyl-protein thioesterase 1 (pdb 1fj2). We found B-factors of the loop as low as the rest of the protein in AtSOBER1, and increased B-factors in the acyl-protein thioesterases APT1 and ZmB6T1C9/ZmAPT2. This indicated that in AtSOBER1 all protein parts retained a similar rigidity, whereas in APT1 and ZmB6T1C9/ZmAPT2 the loop had a higher flexibility than the rest of the protein. To investigate if loop flexibility is directly dependent on the strength of the hydrophobic anchor, we designed a mutant, in which we substituted the phenylalanine, which is the most hydrophobic amino acid on the Hoop-Woods scale, with a leucine (AtSOBER1 F65L). While the mutant crystallized in the same space group and cell dimensions, we found the loop B-factors significantly increased when compared to wild type AtSOBER1 (Fig. 5a–e), indicating that its flexibility had improved just by changing the residue that anchors the loop inside the hydrophobic tunnel. All lid loops examined in this study were exposed to the solvent in their respective crystal structures and they were not included in crystal packing contacts, suggesting that their observed flexibility was of intrinsic nature.

To further examine the importance of the hydrophobic anchor for substrate specificity, we compared Michaelis–Menten parameters between *Arabidopsis thaliana* and *Brassica napus* SOBER1s and TIPSY1s. Like in *Arabidopsis*, the *B. napus* genes are arranged in a tandem. Furthermore, the F65 hydrophobic anchor is conserved between AtSOBER1 and BnSOBER1, while both TIPSY1 proteins carry a leucine residue at the respective position. We discovered a very dominant preference for *p*NP acetate in AtSOBER1 and BnSOBER1, and enhanced performance on longer chains in both TIPSY1 isoforms. We further analyzed mutants in which we swapped the hydrophobic anchor residue between the two *Arabidopsis* proteins. While the catalytic efficiency was decreased in AtSOBER1 by substituting the phenylalanine anchor F65 by a leucine residue, the opposite effect was observed upon replacement of L110 by a phenylalanine residue in AtTIPSY1. We hypothesize that a more flexible lid-loop renders the surface created by L63 in AtSOBER1 less rigid and therefore less suitable for deacetylation. In addition, we found the reverse effect for longer chains. For *p*NP butyrate (C4), valerate (C5), and hexanoate (C6), we measured increased catalytic efficiencies after introducing a weaker anchor (AtSOBER1 F65L) and a decrease in AtTIPSY1 L110F (Fig. 5f, Supplementary Fig. 1d). The observed differences in catalytic efficiencies for those C4–C6 chains are mostly resulting from higher or lower turnover numbers rather than significantly different substrate affinities (Supplementary Table 1). This suggests that the strength of the hydrophobic anchor determines the flexibility of the lid-loop and that a more flexible loop leads to a significant increase in product release.

### Role of SOBER1 in HR repression is conserved in *Brassicaceae*

Since AtSOBER1 and BnSOBER1 show common biochemical characteristics, we tested *Arabidopsis* and *B. napus* isoforms in their ability to suppress AvrBsT-elicited HR in tobacco. Like its *Arabidopsis* homolog, BnSOBER1 was able to repress the hypersensitive response while BnTIPSY failed in doing so (Fig. 5g). This indicated that the biological function of SOBER1 is conserved in the *Brassicaceae* family. Since Xcv is not a *Brassicaceae* pathogen, we tested the effect of AtSOBER1 on HR elicited by two *P. syringae* derived YopJ effectors: HopZ1b and HopZ2. We chose these two effectors since both induce HR in tobacco. In contrast, they were reported to elicit no (HopZ2) or at most very weak (HopZ1b) HR in *Arabidopsis* ^6,23,24^. AtSOBER1 was able to suppress HopZ1b-incited HR, but like AtTIPSY1, had no effect on HR induced by HopZ2 (Supplementary Fig. 5a).

In contrast to *Brassicaceae*, the *Solanaceae* tomato (*Solanum lycopersicum*) and pepper (*Capsicum annuum*) are natural hosts of Xcv. While AvrBsT induces HR in pepper plants, it acts as a virulence factor in tomato^8,25^. Therefore, we tested, if the closest AtSOBER1/AtTIPSY1 homologs from pepper and tomato show different effects on AvrBsT- as well HopZ1b-induced HR in tobacco. Furthermore, we analyzed the closest AtSOBER1/AtTIPSY1 homolog from soybean (*Glycine max*), a host of *hopZ1b*-harboring *P. syringae* strains^6^. All three proteins that we tested failed to suppress HR and they were therefore designated TIPSY1 (Supplementary Fig. 5b–c).

### AtSOBER1 is a protein deacetylase

**Fig. 6:**
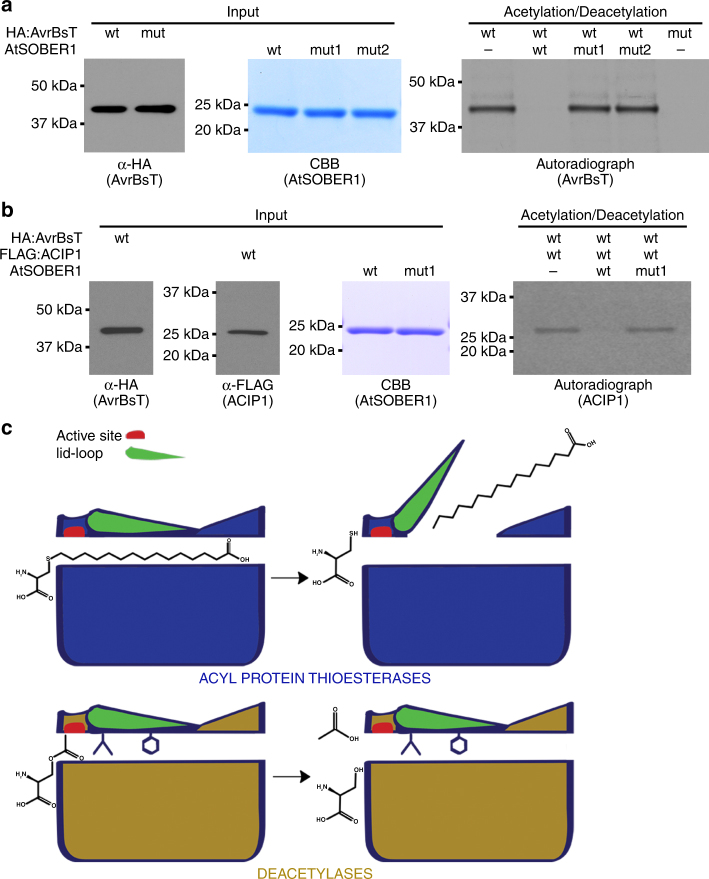
SOBER1 is a protein deacetylase. **a** HA:AvrBsT variants (wild type and catalytic dead mutant C222A) were in vitro translated, affinity enriched and incubated with [^14^C]-acetyl coenzyme A and inositol hexakisphosphate. Subsequent addition of wild type AtSOBER1 deacetylated AvrBsT, while mutant variants (mut1: H192A, mut2: S106A H192A) were inactive. Autoradiography was used to visualize acetylation. (see also Supplementary Fig. 6). **b** FLAG:ACIP1 was in vitro translated, affinity enriched and incubated with in vitro translated HA:AvrBsT, [^14^C]-acetyl coenzyme A and inositol hexakisphosphate. Subsequent addition of wild type AtSOBER1 deacetylated ACIP1, while the mutant variant H192A was inactive. Autoradiography was used to visualize acetylation. **c** Acyl-protein thioesterases feature a long accessible tunnel for accommodation of long-chain fatty acids and release the product by use of a flexible loop that constitutes the tunnel lid. Deacetylases use a hydrophobic residue as anchor to fix the lid and another residue to close the tunnel entrance. This causes an alteration in protein surface and loop rigidity changing the catalytic preference to deacetylation

Like other YopJ effectors, AvrBsT carries acetyltransferase activity^9^. Furthermore, all our structural and biochemical studies of SOBER1 show a strong preference for short acyl chains. We therefore hypothesized that AtSOBER1 might be able to hydrolyze auto-acetylated AvrBsT and its protein substrates. Incubation of in vitro translated AvrBsT in the presence of ^14^C-labeled acetyl-CoA and its cofactor inositol hexakisphosphate (IP_6_) led to auto-acetylation of the wild type protein, but not the enzymatically inactive mutant variant. Subsequent incubation of acetylated AvrBsT with purified AtSOBER1 led to hydrolysis of ^14^C-acetyl-AvrBsT. In contrast, enzymatically inactive AtSOBER1 mutants did not display any protein deacetylation activity (Fig. 6a). Furthermore, longer exposure times revealed that AvrBsT promiscuously trans-acetylated proteins derived from the in vitro transcription and translation system. These proteins were non-specifically bound to the agarose beads used for affinity-purification of AvrBsT and could be visualized via Coomassie staining. Importantly, AtSOBER1 was also able to deacetylate these proteins as promiscuously (Supplementary Fig. 6).

Cheong et al.^9^ showed that ACIP1 is a substrate of AvrBsT and that the ACIP1 family is required for a complete HR induced by AvrBsT. In consequence, AtSOBER1 might suppress AvrBsT-triggered HR by deacetylation of ACIP1. In order to test whether acetylated ACIP1 is a substrate of AtSOBER1, we incubated immobilized ACIP1 with AvrBsT, ^14^C-labeled acetyl-CoA and IP_6_. As described before, this led to trans-acetylation of ACIP1. Subsequently, the acetylated ACIP1 was washed and incubated with purified AtSOBER1. As demonstrated for ^14^C-acetyl-AvrBsT, ^14^C-acetyl-ACIP1 was hydrolyzed by AtSOBER1, but not by its catalytically inactive mutant variant (Fig. 6b).

Altogether, our experiments reveal that AtSOBER1 is a protein deacetylase.

## Discussion

In this work, we have identified a lid-loop as an important structural feature in acyl-protein thioesterases and deacetylases. The lid-loop does not only regulate the substrate turnover rate but also determines protein family and cellular function. We found that a specific arrangement of the loop results in a unique hydrophobic anchor mechanism creating a previously unknown family of protein deacetylases that catalyze the molecular basis for suppression of HR induced by YopJ effector proteins. We furthermore found that SOBER1’s biological function is not restricted to *Arabidopsis thaliana* and not limited to suppress HR induced by AvrBsT. Previous studies identified AtSOBER1 as the enzyme responsible for suppression of *Xanthomonas* AvrBsT-triggered HR in *Arabidopsis* ^16^. Due to contradicting biochemical data and a lack of structural information, the underlying biochemical reaction catalyzed by AtSOBER1 remained questionable. Moreover, the biological significance of AtSOBER1 activity in plants other than *Arabidopsis* and in the context of other YopJ effector proteins was uncertain. We elucidated the catalytic machinery of AtSOBER1, which identified a family of undescribed deacetylases but also revealed important information about the molecular mechanism of acyl-protein thioesterases (Fig. 6c). Acyl-protein thioesterases are present in all domains of life^26^. They feature an active site accessible to the solvent with an adjacent hydrophobic tunnel. Accessibility to the tunnel is crucial for their function and any disturbance can change substrate specificity as shown for the putative third human acyl-protein thioesterase, which turned out to have a function divergent from its paralogs^27,28^. However, acyl-protein thioesterases do not display any obvious lid structure^19^. It remained unclear how substrate specificity and product release would be organized within the protein. In this study, we assessed the structural features that distinguish a previously unknown family of deacetylases from acyl-protein thioesterases, to which the former group has high-sequence identity. We found that a loop constituting the hydrophobic tunnel lid is the major component determining substrate turnover rate and substrate specificity. These findings have direct implications into the catalytic machinery of acyl-protein thioesterases, for which the loop segment turns out to retain the function of a lid. Our study also demonstrates the relative ease with which such lids can be altered to create a different type of hydrolase. The SOBER1 sub-family has incorporated such an alteration that makes deacetylation the preferred reaction catalyzed by the protein. The structural information we obtained allowed us to create a mutant (AtSOBER1 L63A), with impaired ability to deacetylate, but is still a functional enzyme with increased activity on longer substrates. In agreement, this mutant protein is compromised in suppression of HR *in planta*. Taken together with the structural and biochemical data we obtained, this highly suggests deacetylation as the underlying reaction for HR suppression by SOBER1. Our attempts to turn a SOBER1 protein into a TIPSY1 protein by altering the hydrophobic anchor (AtSOBER1 F65L) were a partial success. Weakening the anchor allowed the protein to hydrolyze *para*-nitrophenyl hexanoate, like AtTIPSY1 is able to. AtSOBER1 F65L also displayed higher affinities to longer-chain substrates and the lowest affinity to *para*-nitrophenyl acetate, a pattern opposite to wild type. However, this is not entirely reflected in the catalytic efficiencies due to an almost unaltered turnover rate of *para*-nitrophenyl acetate and, although diminished, AtSOBER1 F65L does not reach the even lower affinity of AtTIPSY1 to *para*-nitrophenyl acetate. Therefore, despite a major contribution of the hydrophobic anchor, we believe that additional protein features exist that mechanistically distinguish SOBER from TIPSY1 proteins, possibly located in the close vicinity of the lid-loop or in the substrate binding site of the proteins. Future studies, including a crystal structure of a TIPSY protein, will hopefully be able to answer this question. Nevertheless, we expect these results to significantly aid the development of small molecule inhibitors for SOBER1 by targeting both the acetate binding site, as well as the loop anchor mechanism inside the hydrophobic tunnel. Such compounds could be used as plant defense supporting molecules, making important crops and biofuel resources carrying *SOBER1* homologs less susceptible to pathogenic bacteria. In addition, as demonstrated by our in vitro and in vivo studies, fine-tuned SOBER1 activity by genetic manipulation could reduce substrate turnover without abolishing enzymatic activity. This study shows that SOBER1’s activity is a significant biological component in HR suppression not only in *Arabidopsis*, but also appears to act in a similar way in the agriculturally important host *Brassica napus*. As shown in vitro, SOBER1 might deacetylate YopJ effectors themselves, preventing a trans-acetylation reaction. Additionally, or alternatively, it might promiscuously deacetylate multiple targets that are acetylated by bacterial effector proteins. SOBER1’s enzymatic activity to counter-react effector proteins is not restricted to AvrBsT, since HR induced by HopZ1b from *Pseudomonas syringae* was successfully suppressed by SOBER1 in our experiments. Additionally, we have shown that SOBER1 deacetylates the microtubule-associated protein ACIP1, which is a substrate of AvrBsT^9^. This shows that SOBER1 directly competes with AvrBsT in a reaction-counterreaction way, and moreover, it seems likely that this competition involves more than one target, given the promiscuity of both AvrBsT and SOBER1. SOBER1’s role might involve the deacetylation of guardee proteins. If a protein guarded by an R protein became acetylated by the acetyltransferase activity of an effector protein, such as AvrBsT, SOBER1’s deacetylation activity would impair target recognition by the R protein and subsequently suppress hypersensitive response. Additional roles of the SOBER1 sub-family remain unclear. Deacetylation is not limited to pathogen infection but it appears disadvantageous to express a too active deacetylase since it suppresses immune response during a bacterial attack. This might be the reason why a weaker anchor is much more common among species than the presence of a very hydrophobic phenylalanine. Modulation of the lid-loop toward a less rigid surface and hence less deacetylation activity might lead to increased fitness against pathogenic bacteria. On the other hand, at certain time points during the evolutionary arms race between bacteria and hosts, it might be advantageous for a plant to express a strong deacetylase. In the absence of an appropriate R protein, its function might serve to counteract the harmful effects of protein acetylation as described for the activity of YopJ on mammalian MAPK kinases and NF-κB pathway kinases^5,29^. Likewise, AvrBsT can act as virulence protein^22,25^. While the presence of ACIP1 and other AvrBsT interactors foster plant defense^30^, SnRK1 was shown to be involved in the virulence function of AvrBsT, the decrease of AvrBs1 elicited HR^22^. Whether acetylation of SnRK1 by AvrBsT is involved in host defense suppression and whether SOBER1 activity would be able to promote HR in this context needs to be addressed by future studies. SOBER1’s reaction mechanism is distinct from known deacetylases. Human histone deacetylases (HDACs) are zinc dependent enzymes and their active site consists of a tyrosine and a charge relay of two histidines and two aspartic acids^31,32^. In addition, class III HDACs are NAD+ dependent^33–35^. These enzymes typically deacetylate lysine residues and in addition, SOBER1 features a classical catalytic triad as active site, comprising a serine, a histidine and an aspartic acid. Therefore, our study suggests that SOBER1 more likely acts as a serine/threonine deacetylase. Altogether, we believe that our results demonstrate a broad significance of SOBER1 function and deacetylation in general for plant immunity. Furthermore, our study is an example how structural data can reveal a protein’s biochemical function that would otherwise stay hidden in bare sequence information.

## Methods

### Molecular cloning

All genes used for bacterial overexpression were synthesized with codon optimization for *E. coli*. Genes were Gateway cloned into a pGEX 4T3 expression vector with a HRV3 protease recognition site immediately upstream to the start codon, leaving two amino acid residues Gly-Pro as cloning artifact. *Arabidopsis thaliana* coding sequences (*AtSOBER1* (*AT4G22305*), *AtTIPSY1* (*AT4G22300*) and *AT5G20060*) used for transient and stable expression were cloned from leaf cDNA; while *BnSOBER1* (*BnaC07g37250D*), *BnTIPSY1* (*BnaC07g37240D*), *CaTIPSY1* (*CA12g00730*), and *SlTIPSY1* (*Solyc11g012070*) were synthesized with codon optimization for *Nicotiana benthamiana* and *GmTIPSY1* (*Glyma17g01270*), *Xanthomonas campestris* pv *vesicatoria avrBsT* and *hopZ1b* (*hopZ1b*_*PgyBR1*_) and *hopZ2* (*hopZ2*_*Ppi895A*_)^6^ were synthesized with codon optimization for *Arabidopsis thaliana*. For transient expression in tobacco leaves, sequences were Gateway cloned into pGWB5 (SOBER1/TIPSY1 family members) or pGWB12 (YopJ effectors)^36^. For in vitro transcription and translation, *avrBsT* was Gateway cloned into pTnT HA:GW^37^. For transformation of *Arabidopsis thaliana* Col-0, *UBQ10*pro in pDONR-P4P1R^38^, CDS of *AtSOBER1* or *AtTIPSY1* in pDONR221 and HA:YFP:HA (DoF tag) in pDONR-P2RP3 were cloned into pH7m34GW via Gateway cloning^39^ (Supplementary Table 2).

### Protein purification

Heterologous expression was carried out in BL21 (DE3), grown at 23 °C until an OD_600_ = 0.6, and induced for 16–18 h at 18 °C using 0.1 mM IPTG. GST fusion proteins were loaded on a glutathione sepharose column in 50 mM TRIS-HCl, 150 mM NaCl, 5% Glycerol, 1 mM TCEP-HCl, final pH 7.7. The column was washed until no protein flow-through could be found by UV detection, and HRV3 protease was added on the column overnight. The cleaved target protein was then eluted using the above buffer, and further purified to homogeneity by size exclusion chromatography using a GE Healthcare HiLoad 16/60 Superdex 75 column in 20 mM TRIS-HCl, 30 mM NaCl, 1 mM TCEP-HCl, final pH 7.7. Proteins were concentrated to at least 10 mg/ml and flash frozen in liquid nitrogen.

### Protein crystallization and structure solution

Protein crystals were grown under the following conditions in 2 µL hanging drops using a 1:1 protein:reservoir ratio. AtSOBER1 wt and AtSOBER1 F65L: 0.1 M MES, pH 6.5, 16% (v/v) PEG 20,000. 25% (v/v) glycerol was used as cryo protectant. AtSOBER1 L63A: 0.1 M HEPES, pH 7.5, 2% (v/v) PEG 400, 2 M ammonium sulfate. 1.14 M Na malonate was used as cryo protectant. ZmB6T1C9: 0.1 M Bis-Tris, pH 5.5, 1% (v/v) PEG 3350, 1 M ammonium sulfate. 1.2 M Na malonate was used as cryo protectant. X-ray data were collected at the Advanced Light Source at Lawrence Berkeley National Laboratory at beamline 8.2.1 at a wavelength of 1.000 Å and at a temperature of 100 K. X-ray data were processed using iMOSFLM^40^ for AtSOBER1 L63A and AtSOBER1 F65L and with XDS^41^ for all other structures. The AtSOBER1 wt and ZmB6T1C9 structures were solved with molecular replacement with PHASER^42^ using human acyl-protein thioesterase 1 (pdb code 1fj2) as template, and all other proteins were then solved with molecular replacement using AtSOBER1 wt as template. Five percent of the data were flagged for R-free and initial models were build using AutoBuild^43^ as part of Phenix^44^, manually corrected and finalized with Coot^45^, refined with phenix.refine^46^, and validated with MolProbity^47^. Structures were refined to R-work/R-free values of 0.169/0.196 (AtSOBER1 wt), 0.188/0.226 (AtSOBER1 L63A), 0.150/0.166 (AtSOBER1 F65L) and 0.183/0.224 (ZmB6T1C9) (Table 1). Ramachandran values were as follows in percent (favored, allowed, outliers): 99.6, 1.4, 0 (AtSOBER1 wt), 99.5, 0.5, 0 (AtSOBER1 L63A), 98.1, 1.9, 0 (AtSOBER1 F65L) and 97.4, 2.6, 0 (ZmB6T1C9). All structures were visualized with CCP4mg^48^.

**Table 1:** Data collection and refinement statistics (molecular replacement)

	AtSOBER1 wild type	AtSOBER1 L63A	AtSOBER1 F65L	ZmB6T1C9/ ZmAPT2
**PDB code**	6AVV	6AVW	6AVX	6AVY
*Data collection*				
Space group	P 2_1_ 2_1_ 2_1_	P 2_1_ 2_1_ 2_1_	P 2_1_ 2_1_ 2_1_	P 2_1_ 2_1_ 2_1_
Cell dimensions				
*a*, *b*, *c* (Å)	45.76, 52.29, 74.23	45.59, 51.55, 76.01	45.50, 52.08, 75.95	48.88, 89.57, 105.21
*α*, *β*, *γ* (°)	90.00, 90.00, 90.00	90.00, 90.00, 90.00	90.00, 90.00, 90.00	90.00, 90.00, 90.00
Resolution (Å)	34.44–1.51 (1.56–1.51)	42.66–2.14 (2.22–2.14)	39.03–1.27 (1.32–1.27)	42.91–2.24 (2.32–2.24)
*R*_sym_ or *R*_merge_	0.08223 (0.759)	0.1427 (0.5133)	0.02519 (0.05704)	0.05712 (0.2155)
*I*/*σI*	21.74 (4.58)	11.31 (5.90)	18.77 (9.09)	14.60 (3.92)
Completeness (%)	100 (100)	100 (100)	98 (95)	93 (72)
Redundancy	13.5 (11.6)	7.4 (6.1)	2.0 (1.9)	3.1 (1.9)
*Refinement*				
Resolution (Å)	34.44–1.51	42.66–2.14	39.03–1.27	42.91–2.24
No. of reflections	28,620	10,288	47,287	21,333
*R*_work_/*R*_free_	0.169/0.196	0.188/0.226	0.150/0.166	0.183/0.224
No. of atoms				
Protein	1630	1610	1621	3487
Water	201	104	311	202
*B*-factors				
Protein	20.26	26.84	11.76	30.99
Water	34.75	33.14	27.76	35.84
R.m.s. deviations				
Bond lengths (Å)	0.005	0.003	0.007	0.003
Bond angles (°)	0.86	0.58	0.95	0.70

*R*_free_ was calculated for 5% of randomly chosen, unique reflections that were excluded from the refinement

Highest resolution shell is shown in parentheses

### Molecular docking of *para*-nitrophenyl esters

Files for molecular docking of *para*-nitrophenyl esters were prepared using PRODRG^49^ and AutoDockTools^50^. Docking was performed with AutoDock Vina^51^. Affinities calculated for *p*NP acetate docked into AtSOBER1 wt were −3.6 kcal/mol and −5.9 kcal/mol for *p*NP palmitate docked into ZmB6T1C9/ZmAPT2. An affinity of −5.0 kcal/mol was calculated for binding of *p*NP valerate into AtSOBER1 L63A.

### Kinetic analysis and phospholipase activity

Parameters of steady-state kinetics were measured using colorimetric compounds (*para*-nitrophenyl (*p*NP) esters) and the release of yellow *p*-nitrophenol was monitored by recording the absorbance at 410 nm at room temperature over 15 min in 60 s intervals using a Tecan Safire II microplate reader. Reactions were measured as triplicates in 20 mM HEPES, 150 mM NaCl, pH 7.52–7.55, 0.01% (v/v) Triton-X100. Enzyme concentrations in the assay were 100 nM. The resulting absorbance was referenced to a linear *p*NP absorbance-concentration relationship and Michelis–Menten parameters were determined with IDBS XLfit. Phospholipase A_2_ activity was measured using the EnzChek Phospholipase A_2_ Assay Kit, in which the release of fluorescent BODIPY from BODIPY-PC-A_2_ was recorded at room temperature at Ex/Em 460/515 nm using a Tecan Safire II microplate reader.

### Protein acetylation and deacetylation assay

According to manufacturer’s instructions, TNT T7 Coupled Reticulocyte Lysate System (Promega) was used to express HA:AvrBsT and FLAG:ACIP1. Reaction mixes were diluted with wash buffer (20 mM Tris, 20 mM NaCl, 1 mM TCEP, 0.1% (v/v) NP-40; pH 7.5) and rotated in the presence of EZview Red Anti-HA Affinity Gel (Sigma) in the case of HA:AvrBsT or with Anti-FLAG M2 Affinity Gel (Sigma) in the case of FLAG:ACIP1 for 1 h at 4 °C. Supernatants were removed and beads were washed six times with wash buffer and three times with acetylation buffer (20 mM Tris, 20 mM NaCl, 1 mM TCEP, 200 nM IP_6_; pH 7.5). For experiments as shown in Fig. 6a, anti-HA beads were incubated for 4 h at room temperature in the presence of 0.5 µCi [Acetyl-1-^14^C]-Acetyl Coenzyme A (PerkinElmer). For experiments as shown in Fig. 6b, anti-FLAG beads were incubated for 4 h at room temperature in the presence of TnT reaction mix containing HA:AvrBsT, 0.5 µCi [Acetyl-1-^14^C]-Acetyl Coenzyme A and 1X acetylation buffer. Afterwards, beads were washed four times with wash buffer, aliquoted and incubated with or without 1 µg AtSOBER1 protein for 2 h. Reactions were stopped by adding sample buffer and boiling. After samples were separated in SDS-PAGEs, gels were stained with Coomassie Brilliant Blue and subsequently dried. X-ray films were incubated from 4 to 7 days. Supplementary Figs. 6 and 7 show the uncropped autoradiographs of Fig. 6a, b.

### Transient expression in *Nicotiana benthamiana*

*Agrobacterium tumefaciens* strain GV3101 was used for transient expression experiments. Overnight cultures were resuspended in infiltration buffer (10 mM MgCl_2_, 10 mM MES, 150 µM acetosyringone; pH 5.6) to OD_600_ of 0.5. For suppression of RNA silencing, an *Agrobacterium tumefaciens* AGL-0 strain harboring p19 was co-infiltrated in all experiments^52^. Tobacco leaves were first infiltrated with bacteria harboring constructs encoding SOBER1/TIPSY1. Twenty-four hours later, the same leaf spot was infiltrated with bacteria transformed with constructs encoding YopJ effectors. Another 24 h later, samples for western blotting were harvested. Two days (for experiments using *avrBsT*) or 3 days (for experiments using *hopZ1b* and *hopZ2*) after the second infiltration photos of leaves were taken. Experiments were carried out three times with six leaves per experiment.

### Western blotting

Frozen tobacco leaf discs were disrupted in a ball mill using glass beads. Ground tissue was boiled in sample buffer and separated in SDS-PAGEs. Following antibodies were used in this study: Anti-HA-Peroxidase, High Affinity clone 3F10 (1:2000, Roche, 11867423001), Anti-FLAG M2-Peroxidase (HRP) Clone M2 (1:5000, Sigma-Aldrich, A8592), anti-GFP clones 7.1 and 13.1 (1:5000, Roche, 11814460001) and Goat Anti-Mouse IgG (H + L)-HRP Conjugate (1:5000, Bio-Rad, 1706516).

### Microscopy

Images were taken with a Zeiss LSM 710 Confocal Microscope. For counterstaining, root tips were incubated in 10 μg/ml propidium iodide for 5 s.

### Data availability

The structural coordinates and diffraction data of the crystal structures have been deposited in the Protein Data Bank under accession codes 6AVV (AtSOBER1 wt), 6AVW (AtSOBER1 L63A), 6AVX (AtSOBER1 F65L) and 6AVY (ZmAPT2). All other data are available from the corresponding author upon reasonable request.

## Electronic supplementary material

Supplementary Information

## References

[1] Galán JE, Collmer A (1999). Type III secretion machines: bacterial devices for protein delivery into host cells. Science.

[2] Büttner D, He SY (2009). Type III protein secretion in plant pathogenic bacteria. Plant Physiol..

[3] Pechous RD, Sivaraman V, Stasulli NM, Goldman WE (2016). Pneumonic plague: the darker side of yersinia pestis. Trends Microbiol..

[4] Amphlett A, Far East (2016). Scarlet-like fever: a review of the epidemiology, symptomatology, and role of superantigenic toxin: yersinia pseudotuberculosis-derived mitogen A. Open Forum Infect. Dis..

[5] Mukherjee S (2006). Yersinia YopJ acetylates and inhibits kinase activation by blocking phosphorylation. Science.

[6] Ma W, Dong FF, Stavrinides J, Guttman DS (2006). Type III effector diversification via both pathoadaptation and horizontal transfer in response to a coevolutionary arms race. PLoS Genet..

[7] Lee AH (2012). A bacterial acetyltransferase destroys plant microtubule networks and blocks secretion. PLoS Pathog..

[8] Minsavage GV (1990). Gene-For-Gene relationships specifying disease resistance in *Xanthomonas campestris* pv. *vesicatoria* – pepper interactions. Mol. Plant Microbe Interact..

[9] Cheong MS (2014). AvrBsT acetylates Arabidopsis ACIP1, a protein that associates with microtubules and is required for immunity. PLoS Pathog..

[10] Jiang S (2013). Bacterial effector activates jasmonate signaling by directly targeting JAZ transcriptional repressors. PLoS Pathog..

[11] Stakman EC (1915). Relation between Puccinia graminis and plants highly resistant to its attack. J. Agric. Res..

[12] Mur LA, Kenton P, Lloyd AJ, Ougham H, Prats E (2008). The hypersensitive response; the centenary is upon us but how much do we know?. J. Exp. Bot..

[13] Abramovitch RB, Martin GB (2004). Strategies used by bacterial pathogens to suppress plant defenses. Curr. Opin. Plant Biol..

[14] Kirik A, Mudgett MB (2009). SOBER1 phospholipase activity suppresses phosphatidic acid accumulation and plant immunity in response to bacterial effector AvrBsT. Proc. Natl Acad. Sci. USA.

[15] Duncan JA, Gilman AG (2002). Characterization of Saccharomyces cerevisiae acyl-protein thioesterase 1, the enzyme responsible for G protein alpha subunit deacylation in vivo. J. Biol. Chem..

[16] Cunnac S (2007). A conserved carboxylesterase is a suppressor of avrbst-elicited resistance in arabidopsis. Plant Cell.

[17] Rocks O (2005). An acylation cycle regulates localization and activity of palmitoylated Ras isoforms. Science.

[18] Ollis DL (1992). The alpha/beta hydrolase fold. Protein Eng..

[19] Devedjiev Y, Dauter Z, Kuznetsov SR, Jones TL, Derewenda ZS (2000). Crystal structure of the human acyl protein thioesterase I from a single X-ray data set to 1.5 A. Structure.

[20] Won SJ (2016). Molecular mechanism for isoform-selective inhibition of acyl protein thioesterases 1 and 2 (APT1 and APT2). ACS Chem. Biol..

[21] Holm L, Rosenström P (2010). Dali server: conservation mapping in 3D. Nucleic Acids Res..

[22] Szczesny R (2010). Suppression of the AvrBs1-specific hypersensitive response by the YopJ effector homolog AvrBsT from Xanthomonas depends on a SNF1-related kinase. New Phytol..

[23] Lewis JD, Abada W, Ma W, Guttman DS, Desveaux D (2008). The HopZ family of Pseudomonas syringae type III effectors require myristoylation for virulence and avirulence functions in Arabidopsis thaliana. J. Bacteriol..

[24] Zhou H, Morgan RL, Guttman DS, Ma W (2009). Allelic variants of the Pseudomonas syringae type III effector HopZ1 are differentially recognized by plant resistance systems. Mol. Plant Microbe Interact..

[25] Kim NH, Choi HW, Hwang BK (2010). Xanthomonas campestris pv. vesicatoria effector AvrBsT induces cell death in pepper, but suppresses defense responses in tomato. Mol. Plant Microbe Interact..

[26] Zeidman R, Jackson CS, Magee AI (2009). Protein acyl thioesterases (Review). Mol. Membr. Biol..

[27] Bürger M (2012). Crystal structure of the predicted phospholipase LYPLAL1 reveals unexpected functional plasticity despite close relationship to acyl protein thioesterases. J. Lipid Res..

[28] Görmer K (2012). Chemical-biological exploration of the limits of the Ras de- and repalmitoylating machinery. Chembiochem: Eur. J. Chem. Biol..

[29] Mittal R, Peak-Chew SY, McMahon HT (2006). Acetylation of MEK2 and I kappa B kinase (IKK) activation loop residues by YopJ inhibits signaling. Proc. Natl Acad. Sci. USA.

[30] Han SW, Hwang BK (2017). Molecular functions of Xanthomonas type III effector AvrBsT and its plant interactors in cell death and defense signaling. Planta.

[31] Vannini A (2004). Crystal structure of a eukaryotic zinc-dependent histone deacetylase, human HDAC8, complexed with a hydroxamic acid inhibitor. Proc. Natl Acad. Sci. USA.

[32] Somoza JR (2004). Structural snapshots of human HDAC8 provide insights into the class I histone deacetylases. Structure.

[33] Avalos JL, Boeke JD, Wolberger C (2004). Structural basis for the mechanism and regulation of Sir2 enzymes. Mol. Cell.

[34] Zhao K, Harshaw R, Chai X, Marmorstein R (2004). Structural basis for nicotinamide cleavage and ADP-ribose transfer by NAD(+)-dependent Sir2 histone/protein deacetylases. Proc. Natl Acad. Sci. USA.

[35] Vanommeslaeghe K (2003). Ab initio study of the binding of Trichostatin A (TSA) in the active site of histone deacetylase like protein (HDLP). Org. Biomol. Chem..

[36] Nakagawa T (2007). Development of series of gateway binary vectors, pGWBs, for realizing efficient construction of fusion genes for plant transformation. J. Biosci. Bioeng..

[37] Nito K, Wong CC, Yates JR, Chory J (2013). Tyrosine phosphorylation regulates the activity of phytochrome photoreceptors. Cell Rep..

[38] Jaillais Y (2011). Tyrosine phosphorylation controls brassinosteroid receptor activation by triggering membrane release of its kinase inhibitor. Genes Dev..

[39] Karimi M, Bleys A, Vanderhaeghen R, Hilson P (2007). Building blocks for plant gene assembly. Plant Physiol..

[40] Battye TG, Kontogiannis L, Johnson O, Powell HR, Leslie AG (2011). iMOSFLM: a new graphical interface for diffraction-image processing with MOSFLM. Acta Crystallogr. D Biol. Crystallogr..

[41] Kabsch W (2010). Xds. Acta Crystallogr. D Biol. Crystallogr..

[42] McCoy AJ (2007). Phaser crystallographic software. J. Appl. Crystallogr..

[43] Terwilliger TC (2008). Iterative model building, structure refinement and density modification with the PHENIX AutoBuild wizard. Acta Crystallogr. D Biol. Crystallogr..

[44] Adams PD (2010). PHENIX: a comprehensive Python-based system for macromolecular structure solution. Acta Crystallogr. D Biol. Crystallogr..

[45] Emsley P, Lohkamp B, Scott WG, Cowtan K (2010). Features and development of Coot. Acta Crystallogr D. Biol. Crystallogr..

[46] Afonine PV (2012). Towards automated crystallographic structure refinement with phenix.refine. Acta Crystallogr D. Biol. Crystallogr..

[47] Chen VB (2010). MolProbity: all-atom structure validation for macromolecular crystallography. Acta Crystallogr D. Biol. Crystallogr..

[48] McNicholas S, Potterton E, Wilson KS, Noble ME (2011). Presenting your structures: the CCP4mg molecular-graphics software. Acta Crystallogr. D Biol. Crystallogr..

[49] Schüttelkopf AW, van Aalten DM (2004). PRODRG: a tool for high-throughput crystallography of protein-ligand complexes. Acta Crystallogr. D Biol. Crystallogr..

[50] Sanner MF (1999). Python: a programming language for software integration and development. J. Mol. Graph Model..

[51] Trott O, Olson AJ (2010). AutoDock Vina: improving the speed and accuracy of docking with a new scoring function, efficient optimization, and multithreading. J. Comput. Chem..

[52] Ripoll JJ (2015). microRNA regulation of fruit growth. Nat. Plants.

