# Supplementary material for: A hydrophobic anchor mechanism defines a deacetylase family that suppresses host response against YopJ effectors
Source: Nat Commun. 2017 Dec 19;8:2201. doi: 10.1038/s41467-017-02347-w (PMC5736716; doi:10.1038/s41467-017-02347-w)
Supplement: Supplementary file 1 — Supplementary Information [file 41467_2017_2347_MOESM1_ESM.pdf]

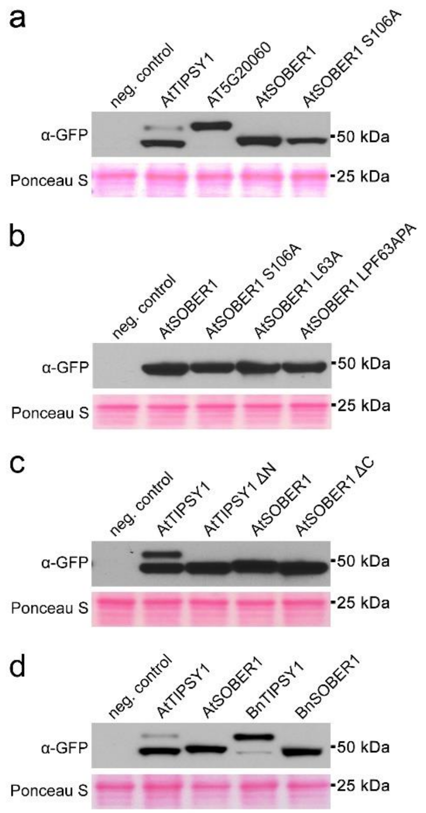

Supplementary Figure 1 | Western blot analysis of proteins expressed in *N. benthamiana* leaves as shown in **Fig. 2**, **Fig. 3**, **Fig. 4** and **Fig. 5**.

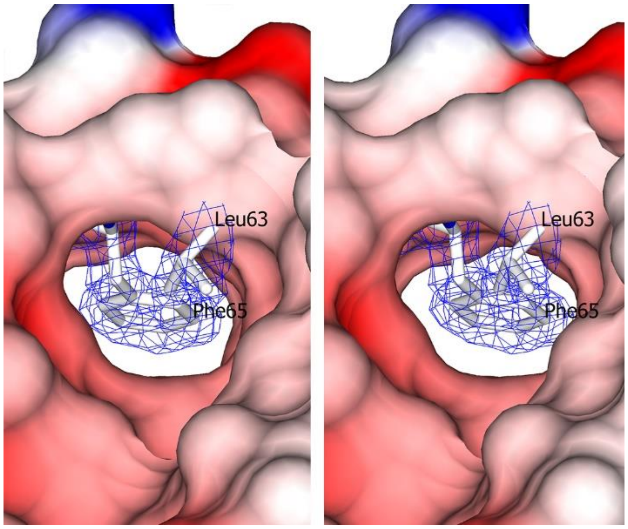

Supplementary Figure 2 | Stereo view of the SOBER1 tunnel with anchor residue Phe65 and residue Leu63 blocking the tunnel entrance. Electron density of Leu63 and Phe65 is shown as 2FOFCWT map contoured at 1 σ.

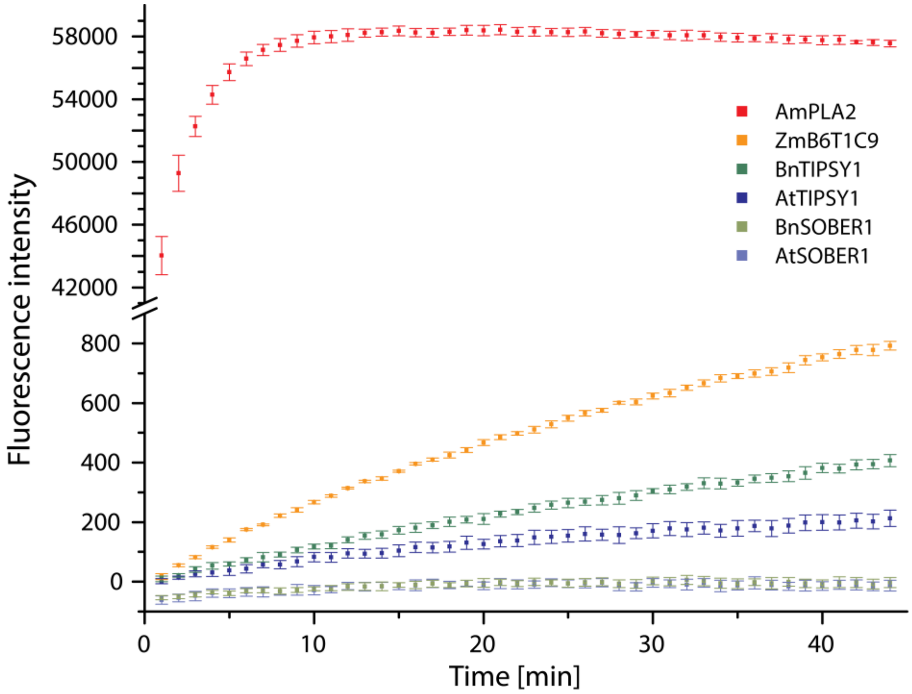

Supplementary Figure 3 | SOBER1 does not display detectable phospholipase A_2 activity.

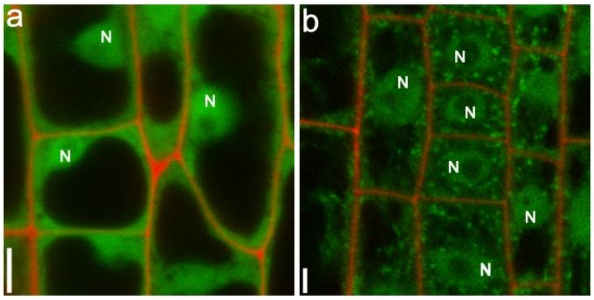

Supplementary Figure 4 | YFP signals of transgenic *Arabidopsis* seedlings expressing *AtSOBER1:HA:YFP:HA* (**a**) or *AtTIPSY1:HA:YFP:HA* (**b**) were detected in nuclei (N) of roots cells. YFP signals are shown in green while propidium iodide is depicted in red. Scale bar = 5 µm.

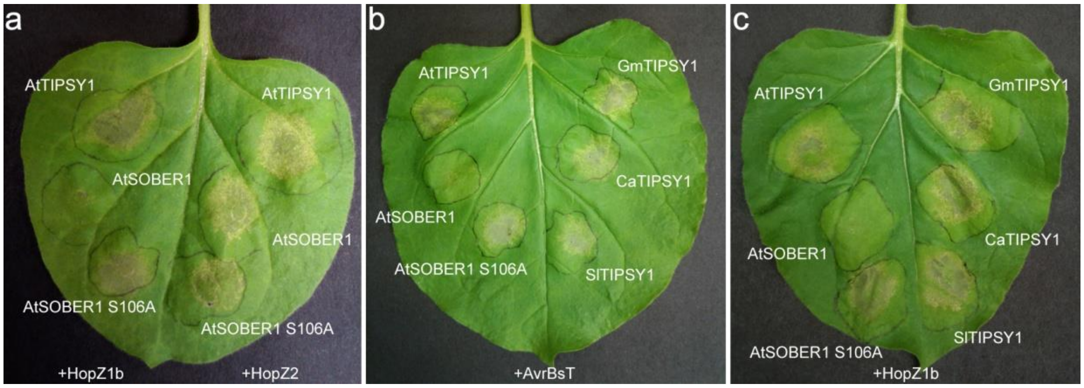

Supplementary Figure 5 | HR elicited by HopZ1b is suppressed by AtSOBER1. (**a**) *Arabidopsis SOBER1* and *TIPSY1* were co-expressed with *hopZ1b* or *hopZ2* from *P. syringae*. (**b**) *AtSOBER1* and *TIPSY1* from *Arabidopsis thaliana*, *Glycine max*, *Capsicum annuum* as well as *Solanum lycopersicum were* co-expressed with *avrBsT*. (**c**) *AtSOBER1* and *TIPSY1* from *Arabidopsis thaliana*, *Glycine max*, *Capsicum annuum* as well as *Solanum lycopersicum were* co-expressed with *hopZ1b*.

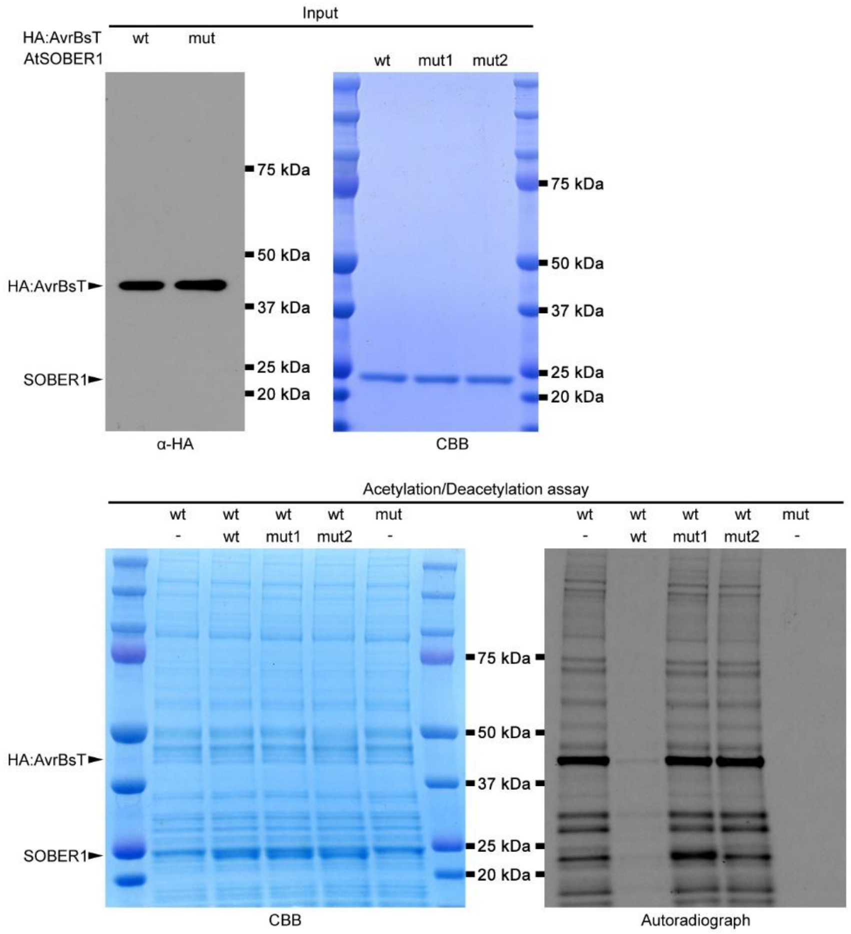

Supplementary Figure 6 | SOBER1 is a protein deacetylase. HA:AvrBsT (wild type and C222A mutant variant) was *in vitro* translated, affinity enriched and incubated with [^{14}C]-acetyl coenzyme A and inositol hexakisphosphate. Subsequent addition of wild type AtSOBER1 deacetylated AvrBsT and other acetylated proteins. These proteins derived from the *in vitro* transcription and translation system and were acetylated by AvrBsT. AtSOBER1 mutant variants (mut1: H192A, mut2: S106A H192A) showed no deacetylation activity.

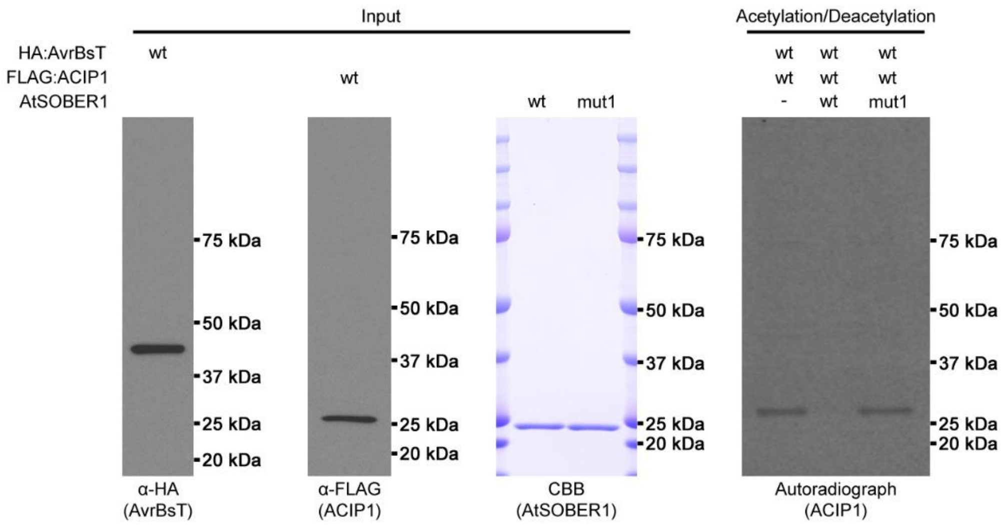

Supplementary Figure 7 | Uncropped scans of western blots, CBB gel and autoradiograph shown in **Fig. 6b**.

Supplementary Table 1 | Kinetic parameters of proteins on different *p*NP esters

	AtSOBER1	AtSOBER1 L63A	AtSOBER1 F65L	AtTIPSY1	AtTIPSY1 L110F
***p*NP acetate (C2:0)**					
K_M [M]	1.58 ± 0.11 × 10^{-4}	6.39 ± 0.22 × 10^{-3}	3.12 ± 0.44 × 10^{-4}	1.75 ± 0.18 × 10^{-3}	1.75 ± 0.33 × 10^{-3}
k_{cat} [s^{-1}]	1.95 ± 0.04 × 10^{2}	3.75 ± 0.09 × 10^{1}	1.88 ± 0.23 × 10^{2}	7.89 ± 0.27 × 10^{2}	4.82 ± 0.32 × 10^{2}
k_{cat}/K_M [$s^{-1}M^{-1}$]	1.24 ± 0.09 × 10^{6}	5.86 ± 0.20 × 10^{3}	6.04 ± 0.84 × 10^{5}	4.51 ± 0.45 × 10^{5}	2.76 ± 0.52 × 10^{5}
***p*NP butyrate (C4:0)**					
K_M [M]	2.27 ± 0.11 × 10^{-4}	2.96 ± 0.15 × 10^{-5}	4.80 ± 0.23 × 10^{-5}	5.03 ± 0.41 × 10^{-4}	4.44 ± 0.82 × 10^{-4}
k_{cat} [s^{-1}]	9.64 ± 0.20 × 10^{0}	1.64 ± 0.03 × 10^{0}	1.10 ± 0.18 × 10^{1}	2.65 ± 0.08 × 10^{2}	1.00 ± 0.07 × 10^{1}
k_{cat}/K_M [$s^{-1}M^{-1}$]	4.25 ± 0.22 × 10^{4}	5.54 ± 0.23 × 10^{4}	2.30 ± 0.37 × 10^{5}	5.10 ± 0.41 × 10^{5}	2.27 ± 0.41 × 10^{4}
***p*NP valerate (C5:0)**					
K_M [M]	3.27 ± 0.27 × 10^{-4}	4.00 ± 0.11 × 10^{-6}	1.52 ± 0.15 × 10^{-4}	3.07 ± 0.29 × 10^{-4}	1.47 ± 0.15 × 10^{-4}
k_{cat} [s^{-1}]	9.01 ± 0.25 × 10^{0}	5.01 ± 0.03 × 10^{0}	1.42 ± 0.06 × 10^{1}	6.46 ± 0.39 × 10^{1}	1.09 ± 0.05 × 10^{1}
k_{cat}/K_M [$s^{-1}M^{-1}$]	2.76 ± 0.22 × 10^{4}	1.25 ± 0.03 × 10^{6}	9.37 ± 0.94 × 10^{4}	2.10 ± 0.20 × 10^{5}	7.39 ± 0.74 × 10^{4}
***p*NP hexanoate (C6:0)**	not determinable	not determinable			
K_M [M]			3.50 ± 0.54 × 10^{-5}	4.75 ± 0.40 × 10^{-4}	1.65 ± 0.15 × 10^{-4}
k_{cat} [s^{-1}]			5.21 ± 0.27 × 10^{0}	3.64 ± 0.18 × 10^{2}	6.28 ± 0.21 × 10^{0}
k_{cat}/K_M [$s^{-1}M^{-1}$]			1.49 ± 0.22 × 10^{5}	7.66 ± 0.64 × 10^{5}	3.80 ± 0.34 × 10^{4}
***p*NP octanoate (C8:0)**	not determinable	not determinable	not determinable	not determinable	not determinable
K_M [M]					
k_{cat} [s^{-1}]					
k_{cat}/K_M [$s^{-1}M^{-1}$]					
***p*NP decanoate (C10:0)**	not determinable	not determinable	not determinable	not determinable	not determinable
K_M [M]					
k_{cat} [s^{-1}]					
k_{cat}/K_M [$s^{-1}M^{-1}$]					
***p*NP dodecanoate (C12:0)**	not determinable	not determinable	not determinable	not determinable	not determinable
K_M [M]					
k_{cat} [s^{-1}]					
k_{cat}/K_M [$s^{-1}M^{-1}$]					
***p*NP myristate (C14:0)**	not determinable	not determinable	not determinable	not determinable	not determinable
K_M [M]					
k_{cat} [s^{-1}]					
k_{cat}/K_M [$s^{-1}M^{-1}$]					
***p*NP palmitate (C16:0)**	not determinable	not determinable	not determinable	not determinable	not determinable
K_M [M]					
k_{cat} [s^{-1}]					
k_{cat}/K_M [$s^{-1}M^{-1}$]					

	BnSOBER1	BnTIPSY1	ZmB6T1C9/ZmAPT2	Ca12g01000	Sl460409648
***p*NP acetate (C2:0)**					
K_M [M]	7.53 ± 0.28 × 10^{-5}	1.19 ± 0.09 × 10^{-3}	1.72 ± 0.43 × 10^{-3}	2.39 ± 0.05 × 10^{-3}	1.43 ± 0.27 × 10^{-3}
k_{cat} [s^{-1}]	7.11 ± 0.09 × 10^{1}	6.34 ± 0.15 × 10^{2}	5.27 ± 0.46 × 10^{2}	1.29 ± 0.01 × 10^{2}	2.32 ± 0.16 × 10^{2}
k_{cat}/K_M [$s^{-1}M^{-1}$]	9.45 ± 0.35 × 10^{5}	2.32 ± 0.37 × 10^{5}	3.07 ± 0.77 × 10^{5}	5.40 ± 0.11 × 10^{4}	1.62 ± 0.31 × 10^{5}
***p*NP butyrate (C4:0)**					
K_M [M]	2.04 ± 0.33 × 10^{-5}	4.70 ± 0.55 × 10^{-4}	5.89 ± 1.26 × 10^{-4}	8.88 ± 0.93 × 10^{-5}	1.93 ± 0.28 × 10^{-4}
k_{cat} [s^{-1}]	2.93 ± 0.16 × 10^{0}	2.22 ± 0.08 × 10^{2}	4.22 ± 0.40 × 10^{2}	8.82 ± 0.26 × 10^{1}	2.18 ± 0.10 × 10^{2}
k_{cat}/K_M [$s^{-1}M^{-1}$]	1.44 ± 0.23 × 10^{5}	4.71 ± 0.55 × 10^{5}	7.17 ± 1.51 × 10^{5}	9.94 ± 0.99 × 10^{5}	1.13 ± 0.16 × 10^{6}
***p*NP valerate (C5:0)**					
K_M [M]	7.83 ± 2.65 × 10^{-5}	3.11 ± 0.32 × 10^{-4}	5.30 ± 0.46 × 10^{-4}	1.38 ± 0.18 × 10^{-4}	1.98 ± 0.35 × 10^{-4}
k_{cat} [s^{-1}]	1.59 ± 0.31 × 10^{0}	1.19 ± 0.05 × 10^{2}	4.41 ± 0.20 × 10^{2}	3.49 ± 0.13 × 10^{1}	1.02 ± 0.07 × 10^{2}
k_{cat}/K_M [$s^{-1}M^{-1}$]	2.03 ± 0.69 × 10^{4}	3.81 ± 0.38 × 10^{5}	8.31 ± 0.07 × 10^{5}	2.51 ± 0.31 × 10^{5}	5.13 ± 0.91 × 10^{5}
***p*NP hexanoate (C6:0)**	not determinable			not determinable	
K_M [M]		1.22 ± 0.10 × 10^{-4}	1.21 ± 0.08 × 10^{-4}		9.64 ± 0.96 × 10^{-5}
k_{cat} [s^{-1}]		1.55 ± 0.05 × 10^{1}	1.39 ± 0.05 × 10^{2}		4.24 ± 0.43 × 10^{0}
k_{cat}/K_M [$s^{-1}M^{-1}$]		1.26 ± 0.10 × 10^{5}	1.59 ± 0.11 × 10^{6}		4.40 ± 0.44 × 10^{4}
***p*NP octanoate (C8:0)**	not determinable	not determinable		not determinable	not determinable
K_M [M]			1.19 ± 0.11 × 10^{-4}		
k_{cat} [s^{-1}]			1.53 ± 0.06 × 10^{2}		
k_{cat}/K_M [$s^{-1}M^{-1}$]			1.28 ± 0.24 × 10^{6}		
***p*NP decanoate (C10:0)**	not determinable	not determinable		not determinable	not determinable
K_M [M]			6.56 ± 0.44 × 10^{-5}		
k_{cat} [s^{-1}]			4.88 ± 0.12 × 10^{1}		
k_{cat}/K_M [$s^{-1}M^{-1}$]			7.43 ± 0.49 × 10^{5}		
***p*NP dodecanoate (C12:0)**	not determinable	not determinable		not determinable	not determinable
K_M [M]			6.40 ± 0.69 × 10^{-5}		
k_{cat} [s^{-1}]			1.07 ± 0.39 × 10^{1}		
k_{cat}/K_M [$s^{-1}M^{-1}$]			1.68 ± 0.18 × 10^{5}		
***p*NP myristate (C14:0)**	not determinable	not determinable		not determinable	not determinable
K_M [M]			3.87 ± 0.33 × 10^{-5}		
k_{cat} [s^{-1}]			1.66 ± 0.07 × 10^{0}		
k_{cat}/K_M [$s^{-1}M^{-1}$]			4.30 ± 0.37 × 10^{4}		
***p*NP palmitate (C16:0)**	not determinable	not determinable		not determinable	not determinable
K_M [M]			9.14 ± 0.10 × 10^{-6}		
k_{cat} [s^{-1}]			6.49 ± 0.06 × 10^{1}		
k_{cat}/K_M [$s^{-1}M^{-1}$]			6.49 ± 0.02 × 10^{4}		

Supplementary Table 2 | List of primers

**Oligos for cloning**	
All constructs for heterologous expression in *E. coli* were synthesized as codon-optimized genes with Gateway cloning sites included so that they could be used without performing PCR. Additionally, coding sequences of *BnSOBER1*, *BnTIPSY1*, *CaTIPSY1*, *GmTIPSY1*, *SlTIPSY1*, *hopZ1b* and *hopZ2* were synthesized with Gateway cloning sites as well.	
In planta experiments:	
ACIP1 3’	GGGGACCACTTTGTACAAGAAAGCTGGGTCTCATCAGTGTATAGAATCTATGTTCTTG
ACIP1 5’	GGGGACAAGTTTGTACAAAAAAGCAGGCTCCATGAAGGAGATGCAGGCAATAG
At5g20060 3’	GGGGACCACTTTGTACAAGAAAGCTGGGTTACCTTCGAGGCTGAGCGTGG
At5g20060 5’	GGGGACAAGTTTGTACAAAAAAGCAGGCTATGAGTATCTCCGGTGCTGC
AtSOBER1 ∆C 3’	GGGGACCACTTTGTACAAGAAAGCTGGGTTACAAGTAGACGACGAACCTTTC
AtSOBER1 3’	GGGGACCACTTTGTACAAGAAAGCTGGGTTGTGGAACATTTCTTTAAGACAATT
AtSOBER1 5’	GGGGACAAGTTTGTACAAAAAAGCAGGCTATGGCTCGAACTTTCATCTTGTGG
AtTIPSY1 ∆N 5’	GGGGACAAGTTTGTACAAAAAAGCAGGCTATGGCTCGAACCTTCATCTTATGG
AtTIPSY1 3’	GGGGACCACTTTGTACAAGAAAGCTGGGTTTGAAGAAGAAGAAGAGCTCTGC
AtTIPSY1 5’	GGGGACAAGTTTGTACAAAAAAGCAGGCTATGCGAACAAGTAGACTGAAGAAGC
avrBsT 3’	GGGGACCACTTTGTACAAGAAAGCTGGGTCTAAGACTCAATAGTCTTTCTGATC
avrBsT 5’	GGGGACAAGTTTGTACAAAAAAGCAGGCTCCATGAAGAACTTCATGAGATCAC
**Mutagenesis oligos**	
Heterologous expression in *E. coli*:	
AtSOBER1 F65L	GCTGGTTTGATGTGCCGGAGCTGCCGCTGAAAGTGGGCAGCCCGATTGACGAGAG
AtSOBER1 H192A	CAAGGCCTATCCGGGCCTGGGCGCGAGCATTAGCAATAAAGAGCTG
AtSOBER1 S106A	GAGCTGGTTTGATATTCCGGAACTGCCGTTTACCGCAGGCAGCCCGAAAGATGAGAGTAG
AtSOBER1 L63A	GCTGGTTTGATGTGCCGGAGGCGCCGTTTAAAGTGGGCAGCCC
AtTIPSY1 L110F	GGAAAACGTGTTCATCTGCGGCCTGGCGCAGGGTGGTGCACTGACCCTGGCAAG
In planta experiments:	
AtSOBER1 L63A	GGTTTGACGTTCCTGAAGCTCCTTTTAAAGTGGGC
AtSOBER1 LPF63APA	GGTTTGACGTTCCTGAAGCTCCTGCTAAAGTGGGCTCTCCAATTGATG
AtSOBER1 S106A	CGTGTTTATCTGTGGATTAGCTCAAGGAGGAGCATTAACC
avrBsT C222A	GCTCAGAAGTCTCTTTTTGATGCTGTTATTTTTTCTTTGAACATG
